# Investigating Bacterial Bloodstream Infections in Dogs and Cats: A 4-Year Surveillance in an Italian Veterinary University Hospital

**DOI:** 10.3390/vetsci12050445

**Published:** 2025-05-06

**Authors:** Raffaele Scarpellini, Massimo Giunti, Cecilia Bulgarelli, Erika Esposito, Elisabetta Mondo, Fabio Tumietto, Silvia Piva

**Affiliations:** 1 Department of Veterinary Medical Sciences, Alma Mater Studiorum—University of Bologna, Via Tolara di Sopra n 50, 40064 Ozzano dell’Emilia, Italy; massimo.giunti@unibo.it (M.G.); cecilia.bulgarelli4@unibo.it (C.B.); erika.esposito6@unibo.it (E.E.); elisabetta.mondo2@unibo.it (E.M.); silvia.piva@unibo.it (S.P.); 2 Unit of Antimicrobial Stewardship, Local Health Authority of Bologna, 40138 Bologna, Italy; fabio.tumietto@ausl.bologna.it

**Keywords:** antimicrobial resistance, veterinary medicine, companion animals, bloodstream infections, blood cultures, antibiotic de-escalation, multidrug resistance

## Abstract

In small animal practice, blood cultures (BCs) are essential for diagnosing bacterial bloodstream infections (BSIs) and guiding targeted antimicrobial therapy, particularly in relation to the rise of multidrug-resistant (MDR) pathogens. This study analyzed 96 positive BCs from dogs and cats at the Veterinary University Hospital (VUH) of Bologna (2020–2024), assessing bacterial prevalence, antimicrobial resistance, and associated risk factors. *Escherichia coli* was the most common isolate (29/96), followed by *Streptococcus canis* (11/96). MDR percentage was 29.2% (28/96), with Gram-negatives associated with higher rates (*p* = 0.040). Nearly half of the cases (46.9%, 45/96) were suspected healthcare-associated infections (HAIs) significantly associated with the number of invasive devices used (*p* = 0.008) and with the absence of co-positive samples (*p* = 0.012). Empirical antibiotic therapy was administered in 94.8% (91/96) of cases, with ampicillin–sulbactam and marbofloxacin as the most used drugs. In vitro empirical therapy appropriateness was 76.9% (70/91). MDR was associated with inappropriate empirical therapy (*p* < 0.001). Mortality within 30 days was 36.5% (35/96), significantly linked to antibiotic escalation (*p* = 0.006). The findings highlight the need for systematic BC surveillance in veterinary settings to optimize treatment strategies (especially in countries with restrictions on antibiotic use in animals) to mitigate MDR spread and to protect public health.

## Simple Summary

This 4-year prospective study investigated bacterial bloodstream infections (BSIs) in dogs and cats hospitalized at an Italian Veterinary University Hospital. From 96 positive blood cultures, *Escherichia coli* was the most common isolate, followed by *Streptococcus canis*. Multidrug resistance (MDR) was observed in 29.2% of cases, mainly among Gram-negative bacteria. Healthcare-associated infections were identified in 46.9% of cases, significantly linked to the use of invasive devices. Empirical antibiotic therapy was initiated in 94.8% of cases, achieving 76.9% in vitro appropriateness; in vitro inappropriate empirical treatment was correlated with MDR. Thirty-day mortality was 36.5%, significantly associated with antibiotic escalation. These findings emphasize the need for continuous blood culture surveillance, targeted antimicrobial stewardship, and infection control measures to optimize therapy and reduce MDR spread in veterinary practice.

## 1. Introduction

Blood cultures (BCs) play a critical role in diagnosing bacteremia and septicemia. These diagnostic tests are frequently used as cost-effective tools to identify the causative agents of bacterial bloodstream infections (BSIs), guiding targeted antimicrobial therapy. BSIs are severe infections that can lead to sepsis, multiorgan failure, and death and can result from a variety of sources, including primary infections, ascending urinary infections, bacterial gut translocation, or healthcare-associated sources such as indwelling catheters or surgical site infections [1]. Compared with human medicine, the management of BSIs in veterinary medicine presents more limitations, such as the lack of advanced monitoring and therapeutics, access to long-term organ support, and financial restrictions [2]. In small animal practice, some of the most common agents associated with bacterial BSIs are multidrug-resistant (MDR) pathogens such as methicillin-resistant *Staphylococcus pseudintermedius* (MRSP), *Pseudomonas aeruginosa*, and extended-spectrum beta-lactamase (ESBL)-producing Enterobacterales [3]. They represent a threat not only for animals but also for public health due to their potential zoonotic transmission. In the European Union, the significant therapeutic challenge they pose is exacerbated by the limitations on antibiotic use that veterinary medicine must deal with [4] in order to preserve the efficacy of the latest antibiotics for human medicine. For these reasons, an accurate and timely isolation and identification of the involved pathogens is crucial not only for effective treatment and infection control but also for mitigating selective pressure with the use of the appropriate antibiotic drug. In this context, the systematic data collection and analysis of BC through surveillance systems represent an effective tool for veterinary healthcare facilities (Morley). The surveillance of BCs allows us to better understand the epidemiology of bacterial BSIs, to detect potential outbreaks early, to monitor and compare antimicrobial resistance (AMR) trends, to promote antimicrobial stewardship, and to optimize the treatment considering local characteristics. The latter is specifically important for BSIs, given that they often require empirical treatment while waiting for the complete diagnosis. Nevertheless, data about BSIs and BCs in veterinary settings are still scarce and not assisted by national or international reference standards. The aims of this study were (i) to describe the prevalence, distribution, and antimicrobial resistance percentage of bacterial pathogenic isolates from positive BCs collected from dogs and cats hospitalized at the Veterinary University Hospital (VUH) of the University of Bologna; and (ii) to determine potential risk factors associated with mortality, healthcare-associated infections, MDR patterns, and inappropriate empirical therapy.

## 2. Materials and Methods

**Study design.**

A perspective, observational, longitudinal study (from January 2020 to December 2024) was conducted at the Veterinary University Hospital of Bologna. Positive BCs obtained from dogs and cats as part of the bacteriological diagnostic routine (*n* = 101) were included in the study.

**BC processing.**

BCs were obtained by aseptically inoculating 5–10 mL of blood into commercial blood culture bottles (Signal Blood Culture System; Oxoid S.p.A., Milan, Italy). Bottles were then incubated at 37 ± 1 °C for a maximum of 7 days. Positive BCs, according to the manufacturer’s instructions, were then plated by streaking in aerobic, capnophilic, and anaerobic conditions at 37 ± 1 °C for 24–47 h. Colonies were macroscopically evaluated, and subsequently, each isolate was identified using the matrix-assisted laser desorption–ionization time-of-flight mass spectrometry method (MALDI-TOF MS) (Biotyper, Bruker Daltonics, Billerica, MA, USA), a rapid and accurate technology for bacterial phenotypic identification, following the manufacturer’s instructions (Bruker Daltonik, Bremen, Germany). The species-level identification was confirmed when the ID score was >2 (green—high accuracy) or >1.8 (for *Staphylococcus* spp. isolates). BCs were considered contaminated and excluded from the analysis after a mutual agreement between the reporting laboratory and the clinical staff, considering both clinical and microbiological findings. Multiple isolates from the same patient obtained from a set of cultures were considered duplicates.

**Antimicrobial susceptibility testing (AST).**

As part of routine laboratory diagnostics, antimicrobial susceptibility testing (AST) was performed with the Kirby–Bauer disc diffusion method, according to the Clinical and Laboratory Standards Institute (CLSI) guidelines [5]. Overall, 15 antimicrobials from 6 antimicrobial classes were included in the final analysis (Table A1). All the discs were purchased from Oxoid (Oxoid S.p.A., Milan, Italy). For every tested drug, each isolate was classified as susceptible (S), intermediate (I), or resistant (R) based on the CLSI veterinary breakpoints [6] or, when not specifically present, based on European Committee on Antimicrobial Susceptibility Testing (EUCAST) breakpoints for human medicine [7]. According to the National Reference Laboratory for AMR [8], antimicrobials known to exhibit expected resistance phenotypes in some bacterial species were not tested. For AST interpretation, intermediate isolates were classified as susceptible, as recommended by the EUCAST [9]. Isolates resistant to at least one drug were considered antimicrobial resistant, and isolates that were not susceptible to at least one antimicrobial drug from three or more antimicrobial classes were considered as non-intrinsic multidrug-resistant (MDR), according to the definition given by Magiorakos et al. [10].

**Data collection.**

For each bacterial isolate, data about the patient signalment (species, age, presence of comorbidities) were extrapolated from the internal laboratory database, as well as hospitalization data when the blood sample was collected (previous hospitalization/surgery in the past 30 days, length of hospitalization, hospitalization in the intensive care unit, surgery, use of invasive devices). Additionally, data about antimicrobial use in the previous 90 days, empirical antimicrobial treatment after BC collection, and antimicrobial treatment after the AST result were recorded. Antimicrobial de-escalation was defined in case of a decrease in the number of antibiotics used or the narrowing of the spectrum of antimicrobial treatment, while escalation was defined in the case of an increase in the number of antibiotics or the broadening of the spectrum, according to the definitions [11,12]. The in vitro empirical appropriateness of the therapy was defined when the bacterial isolate was fully susceptible to at least one of the drugs started empirically. When the specific drug used was not tested, the results from its prototype were used, according to the National Reference Laboratory for AMR [13]. The mortality ratio (natural death or euthanasia) within 30 days of the blood collection was also evaluated. Following the definition by Hacque et al. [14], the suspected healthcare-associated origin of the infection was defined if (i) the blood sample was collected after 48 h or more from the hospitalization and no signs of systemic infection were present at the admission; or (ii) the patient was admitted with signs of systemic infection and had been hospitalized or underwent surgery in the previous 30 days for different reasons.

**Data analysis.**

Descriptive statistics was performed considering bacterial species identified, co-presence of other positive samples (e.g., urines), resistance percentages towards each tested drug and AMR/MDR percentages, anamnestic and hospitalization data, mortality ratio, de-escalation and escalation frequency, in vitro empirical appropriateness of the therapy, and suspected healthcare-associated infection (HAI). Differences in resistance percentages between Gram-positive and Gram-negative bacteria were statistically evaluated using the Fisher exact test, with a *p*-value of <0.05 considered significant. The association between four outcomes (MDR, mortality ratio, appropriateness of the empirical therapy, HAI origin) and all the variables included in the study was evaluated using univariable logistic regression analysis, with a *p*-value of <0.05 considered significant. Normality and heteroskedasticity of data were assessed with the Shapiro–Wilk test and the Levene’s test. Significant results were included in the multivariate analysis model built up with a stepwise selection. Statistical analysis was performed with MedCalc (version v22.009).

## 3. Results

A total of 101 positive blood samples were recorded from 101 patients. The positivity rate was 17.7% (*n* = 101/571). Five samples were excluded from the analysis because the AST was not performed. From the 96 samples included in the study, 22/96 (22.9%) were in co-presence with another positive sample (14 urines, 3 abdominal effusions, 2 uterine swabs, 2 abscesses, 1 synovial liquid). Seventy-four samples (77.1%) were collected from dogs, while twenty-two (22.9%) were from cats. The mean age was 9.4 years (SD 3.8). Sixty-four patients (66.7%) presented at least one comorbidity, of which the most frequent was cancer (*n* = 16), and 35/96 (36.4%) had been hospitalized or underwent surgery in the previous 30 days. Ninety-four patients (98%) were hospitalized when the blood sample was collected, of which seventy-seven (80.2%) were in the Intensive Care Unit and nineteen (19.8%) had surgery. The mean length of hospitalization when blood was collected was 2.5 days (SD 2.3). At least one invasive device was used in 41 patients (57.3%), and a nasogastric tube was the most common (*n* = 35/96, 36.4%). Thirty-six patients (37.5%) were previously treated with antibiotics, including eight cases (8.3%) with more than one. The most common antibiotic class previously used was potentiated penicillins (*n=* 17, 17.7%), followed by fluoroquinolones (*n=* 13, 13.5%).

All the positive BCs were monomicrobial. Ninety-six bacterial isolates were identified, of which 43/96 (44.8%) were Gram-positive, and 53/96 (55.2%) were Gram-negative. The most isolated species were *Escherichia coli* (*n* = 29), *Streptococcus canis* (*n* = 11), and *Staphylococcus pseudintermedius* (*n* = 10) (Table 1). The total MDR percentage was 29.2% (*n* = 28). Non-intrinsic resistance percentages are shown in Table 2. The highest values were recorded for ampicillin (44.7%), tetracycline (43%), and enrofloxacin (29.2%). Gram-negative bacteria were found to be statistically associated with a higher MDR percentage (*p* = 0.040). Forty-five out of ninety-six cases (46.9%) were considered potential HAIs.

An empirical treatment after the BC was started in 91 patients (94.8%). The three most used drugs were ampicillin–sulbactam, empirically started in 51 cases (55.4%), marbofloxacin in 42 (45.6%), and piperacillin–tazobactam in 29 (31.5%). In 40 patients (41.2%), the empirical treatment included more than one drug, with ampicillin–sulbactam/marbofloxacin as the most used combination (*n* = 23 cases). Considering the AST results, empirical antibiotic therapy was considered appropriate in vitro in 70/91 cases (76.9%) (Table 3). The mortality ratio within 30 days was 36.5% (*n* = 35/96). In 24 cases, the patients died before the execution of the AST. After the AST results, antimicrobial treatment was switched in 31/66 (47%) patients, of which 11 (16.7%) were escalations and 20 (30.3%) were de-escalations.

In the multivariate analysis, mortality within 30 days was significantly associated with the escalation of the therapy (*p* = 0.006). Suspected HAIs were associated with the number of invasive devices (*p* = 0.008) and with the absence of co-positive samples (*p* = 0.012). Multidrug resistance was associated with the escalation of the therapy (*p* = 0.012) and with an inappropriate empirical treatment (*p* < 0.001). No direct association between mortality, MDR, and potential HAIs was found.

## 4. Discussion

In this study, we report the results of a 4-year surveillance on positive BCs from dogs and cats admitted to an Italian VUH. BCs are considered the gold standard for diagnosing BSIs [15]. Despite this, studies that specifically provide data from bacterial BCs are lacking in small animal practice. In our research, we registered a positivity rate of 17.7%. Such a value aligns with the few previous veterinary reports, in which it ranged from 20 to 24.4% [16,17,18,19,20]. Positivity rate variability is mainly due to differences in local infection prevalence, diagnostic protocols, or sampling methods. Additionally, the overall accuracy of the test can be affected by the risk of contamination, timing, blood volume, and differences in sampling protocols and techniques [18]. In this sense, the collection of a set of three BCs per patient (instead of one) has been demonstrated to be effective in enhancing sensitivity and specificity [21,22], with increases of the detection rate up to 19% in dogs [23]. In the present study, the direct impact of this diagnostic aspect was not assessed because it was not part of the aims, and it was not systematically performed by clinicians. Nevertheless, when it was executed, it was used as one of the discriminatory factors to exclude potential contaminants [24].

The co-positivity of other samples (mainly urine) for the same bacterial agent is quite a common phenomenon [18,25]. However, it is well established that BCs cannot be replaced by other culture methods, such as urine culture, for the screening or diagnosis of BSIs [26]. The majority of the samples (77.1%) were from dogs, which, compared to cats, tended to be more frequently visited and sampled in the VUH where the study was conducted, as reported by our previous work [27].

The presence of comorbidities was common (66.7%), confirming that BSIs are frequent in patients with other chronic or underlying diseases. Specifically, cancer was the most frequent concurrent disease, remarking that immunocompromised patients, such as oncologic ones, should be specifically monitored, especially in large facilities such as VUHs. The high proportion of patients in this study that were previously hospitalized (36.4%) or hospitalized in the Intensive Care Unit (80.2%) is not surprising, considering that VUHs generally present a conspicuous number of high-risk or referred patients that are more susceptible to pathogens able to cause BSIs, both community-acquired and healthcare-associated (HA BSIs). In detail, we estimated a high frequency of suspected HA BSI (46.9%) in our study. Although this value could have been overestimated due to the assigned definition and the methodology used, it is remarkable to notice that HA BSIs represent an emerging concern for small animal practice. The increasing quality of veterinary healthcare assistance is leading to more attention to the problem but also to a more frequent use of invasive procedures and devices and to a longer length of hospitalization, which are well-recognized risk factors for the development of HAIs [28,29,30]. The mean length of hospitalization when the BC was taken (2.5 days) is in line with this consideration, indicating that in some cases, the BC was not collected or was negative at hospital admission. Similarly, in 57.3% of the patients, at least one invasive device was applied, with the nasogastric tube as the most prevalent. Inadequate food intake during hospitalization is a common issue in companion animals, and nasogastric or esophagostomy feeding tubes are frequently used. If on the one hand, enteral nutrition has been associated with improved outcome in several diseases, such as pancreatitis or septic peritonitis [31,32], on the other hand, the risk of complications, such as tube dislodgement or stoma site or systemic infection, is present [33].

Considering previous antimicrobial use, our reported percentage (37.5%) is in line with previous studies from Europe, highlighting that previous antimicrobial therapy does not necessarily affect BC positivity [18,34]. Potentiated penicillins and fluoroquinolones are confirmed to be the most commonly used drugs in small animal practice, including critical patients [35,36,37,38].

The higher proportion of Gram-negative bacteria (53/96, 55.2%) compared to Gram-positive bacteria (43/96, 44.8%) is consistent with our previous findings [27]. *E. coli* emerged as the most frequently isolated species, followed by *S. canis* and *S. pseudintermedius*. These pathogens are commonly reported as infectious agents in small animal practice, including in bloodstream infections [15,18]. Notably, we observed a relatively high proportion of *Enterobacter* spp. cases associated with a potential HA BSI (*n* = 6, 100%). While three of them were associated with an outbreak, the others were not chronologically close to each other, suggesting the persistence of this pathogen within the hospital. The proportion of other bacteria frequently associated with human BSIs as HAI agents, such as *K. pneumoniae* and *A. baumannii* [39], was instead relatively low. Almost one-third of the isolates (28.9%) were classified as MDR, in line with other reports [18,40]. Gram-negatives were significantly associated with higher MDR percentages (*p* = 0.040). Due to their dynamic structure and their tendency to acquire multiple resistance mechanisms through plasmid conjugation, Gram-negative bacteria such as Enterobacterales develop and disseminate multidrug resistance more frequently [41,42]. This is the main reason why they are predominant in the list of critical pathogens redacted by the WHO and recently updated [43].

We also observed a high percentage of patients in which the empirical treatment was started after the blood collection (95.8%), including 41.2% with two or more different antibiotics. Again, this is not surprising, given that BCs are generally collected in patients in severe conditions, in which empirical treatment is necessary and, if appropriate, lifesaving. A study by Black et al. [38] on 74 dogs admitted to the Intensive Care Unit showed similar results in terms of empirical treatment (94%) and in vitro appropriateness (75%). In our case, the most common drugs used were ampicillin–sulbactam, marbofloxacin, and piperacillin–tazobactam, which had the highest in vitro empirical appropriateness (75.9%). Piperacillin–tazobactam is a potentiated ureidopenicillin categorized as class A (Avoid) by the European Medicines Agency (EMA) [4] and whose use in veterinary medicine had been allowed in the EU until the official prohibition with the 2022/1255 UE regulation. Its regulation is an example of the dilemma that veterinary clinicians are dealing with in Europe. Indeed, while the most powerful antibiotics are restricted or banned for use in animals, the prevalence of pathogens resistant to the allowed drugs is rising, with a consequent narrowing of the treatment options. If on the one hand, this choice can be considered ethical to preserve the most effective and recent antibiotic for human medicine, on the other hand, it implies for veterinarians an increasing risk of therapeutic failure, with all the reputational, economic, and emotional consequences. It is also important to remember that, if overused, important antibiotics such as piperacillin–tazobactam could drive the selection of high-priority pathogens, such as Carbapenem-Resistant Enterobacterales (CRE) [44]. A balanced approach—for example, allowing class A antibiotics only in very specific cases and with an AST to support the decision—could be desirable. Nevertheless, in cases such as severe BSIs where rapid, empirical treatment is needed, this kind of regulation could not be applied. However, compared with piperacillin–tazobactam, a similar in vitro empirical appropriateness (68.6%) was shown by ampicillin–sulbactam, a potentiated aminopenicillin that belongs to the class C (Caution) EMA category, whose use is permitted in animals in the EU. This suggests it could be a good empirical first choice to treat BSIs in small animals, with less risk of MDR selection for public health.

After the AST results, antimicrobial treatment was switched in 31/66 (47%) patients, of which 11 (16.7%) were escalations and 20 (30.3%) were de-escalations. Antibiotic de-escalation is an antimicrobial stewardship strategy aimed at reducing the emergence of AMR and collateral damage from the empirical use of broad-spectrum antibiotics [45]. Nevertheless, its efficacy in reducing MDR rates is still not well weighted [46]. Some authors suggest that ultra-short treatment with no de-escalation in some cases could be more beneficial than unnecessary prolonged treatment with de-escalation [47]. In small animal practices, few studies have assessed the frequency of its application, with ranges from 12% to 63% [16,48]. In our case, we did not detect any association between de-escalation and mortality, inappropriate therapy, or MDR. In contrast, we found a statistically significant association between mortality and escalation (*p* = 0.006) and between MDR and escalation (*p* = 0.012). Antibiotic escalation is normally executed when the pathogen shows multiple resistances or, in the case of non-responsive patients, with an already compromised condition that often, despite the antibiotic shift, leads to death. This could suggest that in most cases, antibiotic escalation does not furnish any benefit. To avoid unnecessary shifts, a study from human medicine proposed the use of the “Escalation Antibiogram”, a model to inform empiric treatment changes in nonresponsive patients, using data from Gram-negative BSIs [49].

Notably, we did not find any correlation between mortality and MDR, in vitro appropriateness of the therapy, and HAIs. Although this finding could be due to the relatively small sample size, other studies in small animal practice described similar results [2,40,50,51,52]. Indeed, mortality is influenced by a plethora of other factors, such as the patient’s response, bacterial load, therapy timing, route of administration, and doses [2,53]. On the other hand, we found a strong correlation between MDR and inappropriate therapy, which can be easily explained by the fact that bacteria resistant to multiple drugs are more likely empirically treated with one ineffective agent. Similarly, the association between potential HA BSIs and the number of invasive devices (*p* = 0.008) finds its explication in the fact that invasive devices, such as nasogastric tubes or indwelling catheters, are the main route of HAI infections [14]. For this reason, they should be removed as soon as possible and managed with caution and an evidence-based approach. For example, in human medicine, regular intravenous catheter substitutions are not recommended [54,55].

Additionally, we found that the patients with co-positive samples were less likely to develop suspected HA BSIs (*p* = 0.012). The presence of another positive sample (e.g., urine) for the same pathogen indicates that the BSI was probably a consequence of the expansion of the infection from the site of origin (e.g., the urinary tract). Conversely, HA BSIs are often caused by bacteria that directly infect the bloodstream through indwelling catheters or other invasive devices, without the involvement of other districts.

This study has some limitations. First, the sample size was relatively small, affecting the statistical power of the analysis. Second, the in vitro appropriateness of the therapy did not consider many other pharmacokinetics and clinical factors (dose, duration, timing) that could have influenced the efficacy of the treatment. Third, the methodology used to perform the AST (Kirby–Bauer) was semi-quantitative, with a subsequent loss in the sensitivity and accuracy of the test.

## 5. Conclusions

In conclusion, this research aimed to add novelty to the prevalence, the resistance rates, and the associated factors of bacterial BSIs in small animal medicine. Our findings emphasize the need for surveillance plans, antimicrobial stewardship programs, and improved infection control protocols in companion animals. While the progress made by human medicine should be used as a reference, the specific differences, such as the limitations on antibiotic use, should also be considered to optimize BSI management in this field. This is specifically important considering the deep interconnection between animal and human health and the risk of interspecies transmission.

## Figures and Tables

**Table 1:** Distribution of the 96 analyzed isolates based on bacterial species identification, and the number of multidrug-resistant (MDR) isolates for each species.

Bacterial Species	Total Isolates (%)	Number of Non-Intrinsic MDR Isolates (%)
*E. coli*	29 (30.2)	11 (37.9)
*Enterobacter* spp.	6 (6.3)	4 (66.7)
*Enterococcus* spp.	4 (4.2)	0 (0)
*S. canis*	11 (11.5)	0 (0)
*S. gallolyticus*	4 (4.2)	0 (0)
*K. pneumoniae*	4 (4.2)	3 (75)
other Gram-negatives	8 (8.3)	2 (25)
*S. pseudintermedius*	10 (10.4)	5 (50)
*S. aureus*	5 (5.2)	1 (20)
other Gram-positives	10 (5.2)	2
*P. aeruginosa*	5 (5.2)	0

**Table 2:** Resistance percentages of the 96 isolates included in the study for each tested antibiotic. Overall results are shown, as well as results of Gram-positive and Gram-negative isolates. The *p*-value to evaluate the differences between Gram-positives and Gram-negatives obtained with Fisher’s exact test is shown. Values considered significant (*p* < 0.05) are in bold and marked with *.

Antibiotic Drug	n. of Tested Isolates	n. of Non-Intrinsic Resistance (%)	n. of Resistant Gram-Positive Isolates (%)	n. of Resistant Gram-Negative Isolates (%)	*p*-Value
Amikacin	72	0 (0)	0/17 (0)	0/57 (0)	NA
Gentamicin	76	8 (10.5)	3/24 (12.5)	5/52 (9.6)	0.983
Ampicillin/Penicillin G (for *Staphylococcus* spp. isolates)	76	34 (44.7)	17/43 (39.5)	17/33 (51.5)	0.297
Oxacillin/Cefoxitin (for *Staphylococcus* spp. isolates)	19	7 (36.8)	NA	NA	NA
Amoxicillin–clavulanic acid	81	21 (25.9)	9/43 (20.9)	12/38 (31.6)	0.275
Ampicillin–sulbactam	81	18 (22.2)	8/43 (18.6)	10/38 (26.3)	0.405
Piperacillin–tazobactam	96	12 (12.5)	8/43 (18.6)	4/53 (7.5)	0.103
Cefazolin	76	23 (30.3)	8/38 (21.1)	15/38 (39.5)	0.080
Ceftiofur	86	23 (26.7)	8/39 (20.5)	15/47 (31.9)	0.139
Ceftazidime (for *P.aeruginosa* isolates)	6	0(0)	NA	NA	NA
Tetracycline	93	40 (43)	19/43 (44.2)	21/50 (42)	0.832
Enrofloxacin	96	28 (29.2)	9/43 (20.9)	19/53 (35.8)	0.110
Trimethoprim–sulfamethoxazole	89	23 (25.8)	7/41 (17.1)	16/48 (33.3)	0.081
Non-intrinsic multidrug resistance	96	28 (29.2)	8/43 (18.6)	20/53 (38)	0.040 *

**Table 3:** Distribution of empirical antibiotic treatment in the 96 patients included in the study according to the antibiotic used. The number of cases in which the antibiotic was considered in vitro appropriate is also shown.

Antibiotic	n. of Patients Empirically Treated	In Vitro Appropriateness	%
Ampicillin–sulbactam	51	35	68.6%
Marbofloxacin	42	18	42.9%
Piperacillin–tazobactam	29	22	75.9%
Other	6	1	16.7%
Total	91	70	76.9%

## Data Availability

The raw data supporting the conclusions of this article will be made available by the authors on request.

## Abbreviations

The following abbreviations are used in this manuscript:

BC	Blood culture
BSI	Bloodstream infection
AMR	Antimicrobial resistance
MDR	Multidrug resistance
HAI	Healthcare-associated infection
VUH	Veterinary University Hospital
EUCAST	European Committee on Antimicrobial Susceptibility Testing
CLSI	Clinical and Laboratory Standards Institute
AST	Antimicrobial susceptibility testing

## Appendix A

**Table A1:** List of tested antimicrobials divided by antimicrobial class.

Antimicrobial Class	Antimicrobial Drug
Aminoglycosides	Amikacin 30 μg
	Gentamicin 10 μg (120 μg for *Enterococcus* spp. isolates)
Penicillins +/− beta-lactamase inhibitors	Ampicillin 10 μg
	Penicillin G 10 units (for *Staphylococcus* spp.)
	Oxacillin 1 μg (for *Staphylococcus pseudintermedius* and *Staphylococcus schleiferi)*
	Amoxicillin–clavulanate 30 μg
	Ampicillin–sulbactam 20 μg
	Piperacillin–tazobactam 110 μg
Cephalosporins	Cefazolin/cephalothin 30 μg
	Cefoxitin 30 μg (for *Staphylococcus* spp. other than *S.pseudintermedius* and *S.schleiferi*)
	Ceftiofur 30 μg
	Ceftazidime 30 μg (for *Pseudomonas aeruginosa*)
Tetracyclines	Tetracycline 30 μg
Fluoroquinolones	Enrofloxacin 5 μg
Sulfonamides + dihydrofolate reductase inhibitors	Trimethoprim–sulfamethoxazole 1.25/23.7 μg
